# Self-Assembly of Discrete Metal Complexes in Aqueous Solution via Block Copolypeptide Amphiphiles

**DOI:** 10.3390/ijms14012022

**Published:** 2013-01-21

**Authors:** Keita Kuroiwa, Yoshitaka Masaki, Yuko Koga, Timothy J. Deming

**Affiliations:** 1 Department of Nanoscience, Faculty of Engineering, Sojo University, 4-22-1 Ikeda, Nishi-ku, Kumamoto 860-0082, Japan; E-Mails: 1114m07@gmail.com (Y.M.); 1214m03@gmail.com (Y.K.); 2 Department of Bioengineering, University of California, Los Angeles, CA 90095, USA; E-Mail: demingt@seas.ucla.edu

**Keywords:** self-assembly, metal complexes, nanostructure, photoluminescence, metal-metal interaction, nanorod, block copolypeptides, amphiphiles

## Abstract

The integration of discrete metal complexes has been attracting significant interest due to the potential of these materials for soft metal-metal interactions and supramolecular assembly. Additionally, block copolypeptide amphiphiles have been investigated concerning their capacity for self-assembly into structures such as nanoparticles, nanosheets and nanofibers. In this study, we combined these two concepts by investigating the self-assembly of discrete metal complexes in aqueous solution using block copolypeptides. Normally, discrete metal complexes such as [Au(CN)_2_]^−^, when molecularly dispersed in water, cannot interact with one another. Our results demonstrated, however, that the addition of block copolypeptide amphiphiles such as K_183_L_19_ to [Au(CN)_2_]^−^ solutions induced one-dimensional integration of the discrete metal complex, resulting in photoluminescence originating from multinuclear complexes with metal-metal interactions. Transmission electron microscopy (TEM) showed a fibrous nanostructure with lengths and widths of approximately 100 and 20 nm, respectively, which grew to form advanced nanoarchitectures, including those resembling the weave patterns of *Waraji* (traditional Japanese straw sandals). This concept of combining block copolypeptide amphiphiles with discrete coordination compounds allows the design of flexible and functional supramolecular coordination systems in water.

## 1. Introduction

There has, to date, been significant interest in the design and fabrication of low dimensional metal complexes, primarily because the electronic structures of such complexes are tunable via the formation of supramolecular architectures such as nanoparticles [1–19], nanocrystals [20–23] and nanowires [24–35]. The realization of dynamic correlations between molecular structures and supramolecular structures remains the most significant challenge in developing functional materials, which exhibit charge transfer, spin state crossover, photoluminescence and other useful properties. The luminescent properties of nanoscale metal complexes such as nanowires and nanocrystals are known to be especially sensitive to molecular structure, molecular conformation, metal-metal interactions and nanostructure [25,26,36,37]. The *d*^10^ gold(I) complexes, in particular, aggregate through *d*^10^–*d*^10^ closed shell aurophilic bonding interactions, which determine both the supramolecular structures and luminescent properties of these materials [38,39].

As a result of its dynamic luminescence properties, [Au(CN)_2_]^−^ has applications as a functional material within intelligent molecular systems. Both the wavelength and intensity of this luminescent emission can be tuned based on the aggregation of [Au(CN)_2_]^−^ through Au-Au bonding interactions, although high concentrations (>10 mM) are required for luminescence at ambient temperatures [40–42]. At present, however, the relationships between molecular structure, metal-metal interactions and morphology at the nanoscale level are not thoroughly understood with regard to their effects on supramolecular structure, although simple polymers can lead to the polyelectrolyte with [Au(CN)_2_]^−^ and self-assembly of polymeric [Au(CN)_2_]^−^ [43]. The ability to tune the nanoscale morphology of these materials via molecular structure could lead to dramatic advances in the functionalities of systems incorporating metal complexes, and enable the dynamic hierarchical structural transformations, which lie at the very heart of bottom-up nanotechnology.

Diblock copolypeptide amphiphiles are synthetic materials with many features that make them of interest to those working in the field of protein engineering, in applications such as drug delivery systems and tissue engineering [44–50]. Their unique properties are due to the propensity of these amphiphiles to form double-walled vesicles or biocompatible fibrillar nanostructures based on the self-assembly of their hydrophilic and hydrophobic blocks. Moreover, the resulting hierarchical microstructures are suitable for integration with organic molecules, tissue cells and proteins. In addition, these copolypeptide structures can be used to tune various inorganic materials. They may, for example, be employed to modify the molecular structure, porosity and morphology of silica [51,52]. The inherent functional self-assembly abilities of these copolypeptide amphiphiles could potentially lead to their application not only as structural templates for inorganic compounds but also for the intelligent transformation of such inorganic materials involving dynamic tuning of the electronic state of the material.

In this study, we focus on the dynamic structural transformation of [Au(CN)_2_]^−^, achieved through the use of diblock copolypeptide amphiphiles having the general structural formula poly-l-Lysine-block-l-Leucine (K_m_L_n_). These amphiphiles are known to assemble into fibrillar structures, resulting in the formation of hydrogels and vesicles [52–55]. Our work investigated not only the morphological evolution associated with the aurophilic and polymeric interactions of the [Au(CN)_2_]^−^, but also the hierarchical transformations of composite materials composed of combinations of the copolypeptides with the Au complex. The nature of the systematic assembly of these materials in solution is discussed, based on the results of spectroscopic and microscopic measurements.

## 2. Results and Discussion

### 2.1. Preparation of Copolypeptides

**Figure 1:**
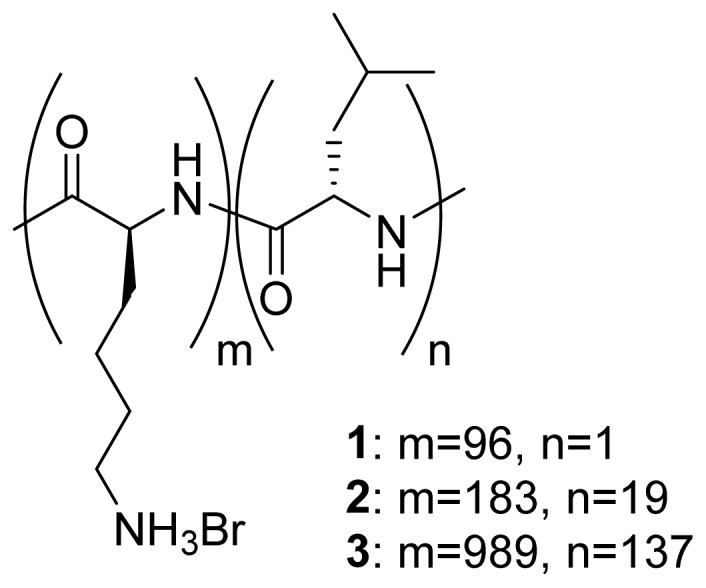
Chemical structures of the diblock copolypeptide amphiphiles K_96_L_1_ (**1**), K_183_L_19_ (**2**) and K_989_L_137_ (**3**).

A number of diblock copolypeptide amphiphiles were synthesized according to procedures previously published in the literature [44,45]: K_96_L_1_ (**1**), K_183_L_19_ (**2**) and K_989_L_137_ (**3**) (Figure 1). All three copolypeptides exhibited low polydispersity values, ranging from 1.13 to 1.19, as measured by gel permeation chromatography (GPC) and ^1^H NMR integration data for the lysine (Lys) moieties. When these copolypeptide amphiphiles were dissolved in water at concentrations above 1 wt%, hydrogels were obtained. This result indicates that all the synthesized copolymers formed fibrillar structures in aqueous solution as a result of self-assembly of the Lys and leucine (Leu) segments of the amphiphilic molecular structure.

### 2.2. Spectroscopic Properties of Amphiphile/Metal Complex Composites

**Figure 2:**
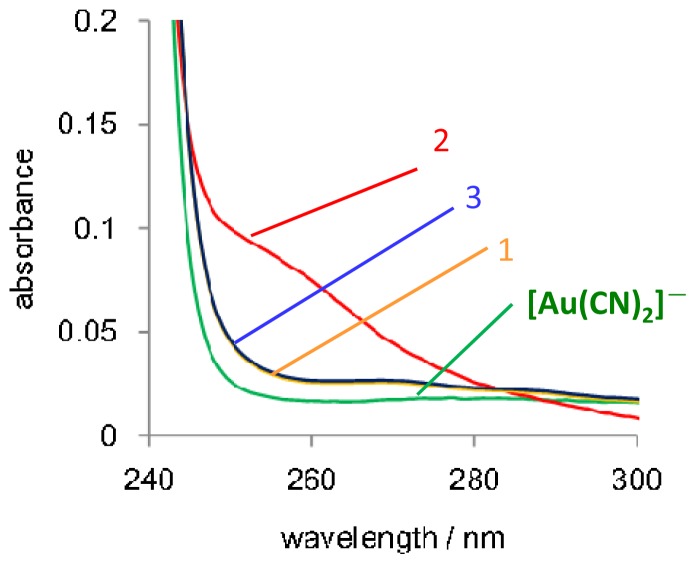
UV-vis absorption spectra of composites **1**/[Au(CN)_2_]^−^, **2**/[Au(CN)_2_]^−^ and **3**/[Au(CN)_2_]^−^ in deionized (DI) water. [Au(CN)_2_]^−^ = 0.2 mM (Lys units:K[Au(CN)_2_] = 1:1).

When K[Au(CN)_2_] was added to aqueous solutions of the diblock copolypeptide amphiphiles at 1:1 molar ratios of [Au(CN)_2_] to lysine unit, dispersion solutions were obtained. The associated aggregation and self-assembly of [Au(CN)_2_]^−^ were investigated by UV-vis spectroscopy (Figure 2). The addition of 0.2 mM DI water solutions of K[Au(CN)_2_] to diblock copolypeptide amphiphiles **1**–**3** resulted in the appearance of a new absorption shoulder in the region of 250 to 280 nm. Interestingly, the absorbance in this region was found to increase in the following order: **2**/[Au(CN)_2_] > **3**/[Au(CN)_2_] > **1**/[Au(CN)_2_]. These observations suggest the oligomerization and/or polymerization of [Au(CN)_2_]^−^ based on both electrostatic interactions with the positively charged side groups of the amphiphile Lys moieties and aurophilicity. Typically, when combining polylysine (without a Leu component) with K[Au(CN)_2_], an increase in the Lys to K[Au(CN)_2_] ratio results in a gradual increase in the absorbance of the shoulder at 250 to 300 nm [43]. It is therefore noteworthy that the copolypeptide amphiphiles in this work were synthesized to a degree of polymerization such that they were well suited to self-assembly and also were able to form structures, which promoted aurophilic interactions. In the case of these particular amphiphiles, it seems that not only their molecular structure and amphiphilic sequence, but also the degree to which they are polymerized has an effect on the aurophilic interactions of the metal complex composite.

**Figure 3:**
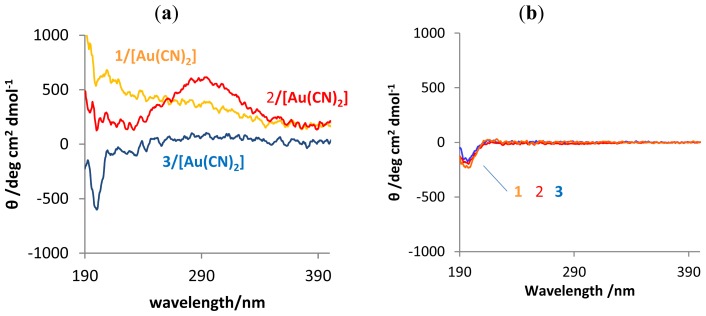
(**a**) Circular dichroism (CD) spectra of **1**/[Au(CN)_2_]^−^, **2**/[Au(CN)_2_]^−^ and **3**/[Au(CN)_2_]^−^ in DI water. [Au(CN)_2_]^−^ = 0.2 mM. (Lys units:K[Au(CN)_2_] = 1:1). (**b**) CD spectra of **1**, **2** and **3** in DI water ([Lys unit] = 0.2 mM).

The circular dichroism (CD) spectra used to determine the conformations of the amino acids in water are presented in Figure 3a. In the composite mixtures, induced circular dichroism (ICD) appears in the absorbance region of 250 to 300 nm associated with [Au(CN)_2_]^−^, and the signal corresponding to such conformation as α-helix or β-sheet was not observed. The results indicate the assembly of [Au(CN)_2_]^−^ anions around the segments of the amphiphile backbone containing the cationic Lys, and a concurrent arrangement in the polymer conformation of random coil. In general, the CD spectra of diblock copolypeptide amphiphiles with Leu segment (greater than 20 residues) in DI water solutions at 293 K indicate an α-helix conformation due to hydrogen bonding interactions between the amino acids and hydrophobic interactions among the Leu segments [44,45]. On the other hand, **1**, **2**, and **3** cannot lead to the special secondary conformation due to much longer Lys segments (Figure 3b). Therefore, this result suggests that aurophilic and electrostatic interactions between the Lys segments and the [Au(CN)_2_]^−^, as well as hydrophobic interaction between the Leu segments, induced a random backbone.

It is well known that the specific conformations of protein molecules, such as α-helix, β-sheet or random coil, are closely associated with biological activity and play an important role in the functioning of the protein. Based on our general knowledge of polypeptides, the stabilization of a polypeptide’s β-sheet structure using negatively charged metal complexes should be possible [43]. In the case of this study, however, interactions of the anionic metal complex with the Lys segments of the copolypeptide amphiphiles induces conformational changes that transform the polymeric backbone to a random coil which in turn allows aurophilic interactions.

**Figure 4:**
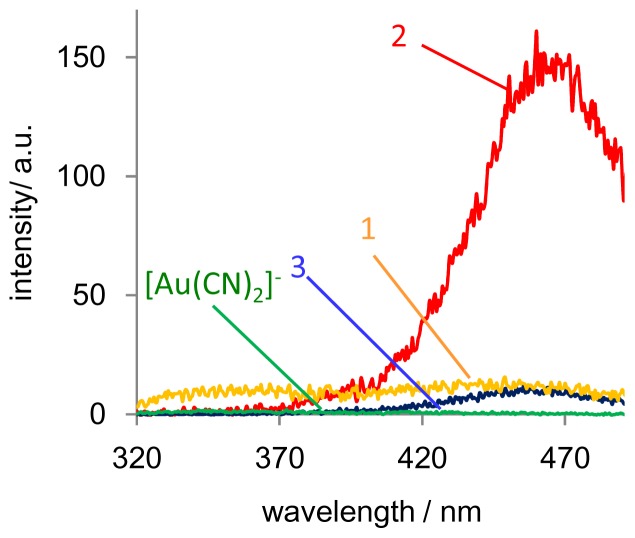
Fluorescence spectra of **1**/[Au(CN)_2_]^−^, **2**/[Au(CN)_2_]^−^ and **3**/[Au(CN)_2_]^−^ in DI water. [Au(CN)2]^−^ = 0.2 mM. (Lys units:K[Au(CN)_2_] = 1:1).

**Figure 5:**
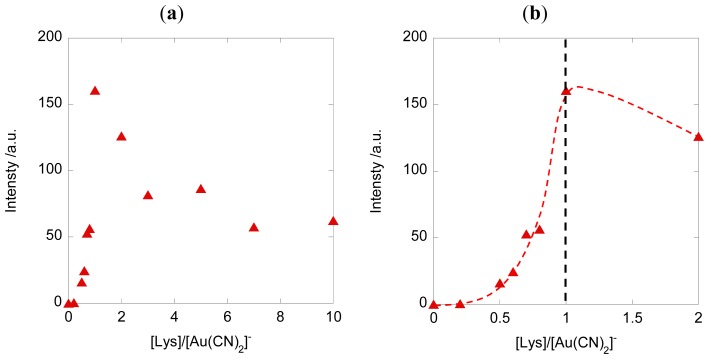

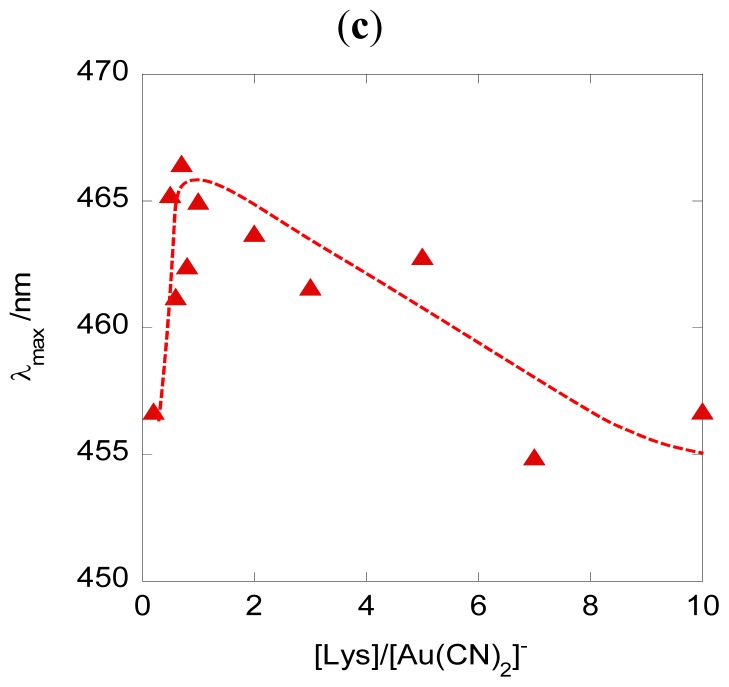
(**a**,**b**) Variations in the fluorescent intensity of **2**/[Au(CN)_2_]^−^ with changes in the lysine:[Au(CN)_2_]^−^ molar ratio (a: 0–10, b: 0–2) in DI water. (**c**) Variations in the fluorescence wavelength of **2**/[Au(CN)_2_]^−^ with changes in the lysine:[Au(CN)_2_]^−^ molar ratio in DI water. [Au(CN)_2_]^−^ = 0.2 mM.

**Figure 6:**
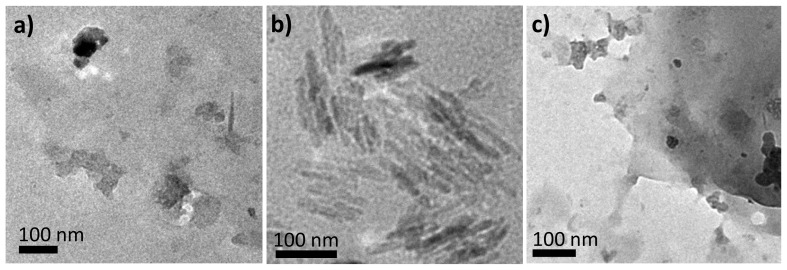
Transmission electron micrographs of samples prepared from (**a**) **1**/[Au(CN)_2_]^−^, (**b**) **2**/[Au(CN)_2_]^−^ and (**c**) **3**/[Au(CN)_2_]^−^ in DI water solutions. Samples are not stained.

We also investigated the aggregation of [Au(CN)_2_]^−^ and its aurophilic interactions by obtaining luminescence spectra of the amphiphile/[Au(CN)_2_]^−^ composites. The excitation and emission spectra of composites **1**–**3**/[Au(CN)_2_]^−^ (Lys units:K[Au(CN)_2_] = 1:1) are shown in Figure 4. A 0.2 mM solution of [Au(CN)_2_]^−^ in the absence of the copolypeptide did not exhibit luminescence. In the case of a 2 mM [Au(CN)_2_]^−^ solution, however, luminescence was observed at 343 nm (ex. 260 nm), a result which has previously been reported and attributed to the [Au(CN)_2_]^−^ trimer [41,42]. The intensity of the luminescence increased following the addition of amphiphiles **1** to **3**. Amphiphile **2** in particular resulted in a dramatic increase in luminescence intensity (Figure 5). The effect of varying the stoichiometric ratio of the composite solution was also investigated, based on the addition of **2** to a 0.2 mM [Au(CN)_2_]^−^ solution in deionized (DI) water. The addition of **2** (0.2 to 1 molar equivalents of the Lys unit) to K[Au(CN)_2_] induced a sigmoidal increase of the emission intensity, as well as a red shift of the emission band to 465 nm (ex. 271 nm) (Figure 6). Further addition of **2** (1 to 10 molar equivalents of the Lys unit) led to a decrease in the emission intensity with a slight blue shift of the emission band to 456 nm (Figure 5). It has been reported that the luminescence band resulting from the Au-Au bond may be attributed to polynuclear [Au(CN)_2_^−^]*_n_* excimers and exciplexes [41,42]. Furthermore, the red-shifted emission band (seen upon adding 0.2 to 1 molar equivalents Lys) indicates longer polynuclear Au complexes than were present in the initial solution of the complex, whereas the blue shift (at 1 to 10 molar equivalents Lys) suggests a decrease in polynuclear species due to disordered Au–Au interactions (Figure 5). In contrast, amphiphile **1** (which had a lower extent of polymerization than **2**) and amphiphile **3** (which had a greater extent of polymerization than **2**) only caused slight increases in luminescent intensity. These results indicate that electrostatic and van der Waals interactions with the amphiphiles play a significant role in the luminescence resulting from the Au-Au interactions. Based on these studies concerning the self-assembly of [Au(CN)_2_]^−^ with the diblock copolypeptide amphiphiles, we conclude that the aurophilic interaction is readily controlled via both the concentration of the metal complex in solution and the molar ratio between the peptide and the complex.

### 2.3. Morphological Characterization of Amphiphile/Metal Complex Composites

To examine the detailed nanostructures of the amphiphile/[Au(CN)_2_]^−^ complexes, transmission electron micrographs of DI water solutions of **1**/[Au(CN)_2_]^−^, **2**/[Au(CN)_2_]^−^ and **3**/[Au(CN)_2_]^−^ were acquired (Figure 6). These samples were not stained, and the dark regions are ascribed to Au present in the composites. Both **1**/[Au(CN)_2_]^−^ (Figure 6a) and **3**/[Au(CN)_2_]^−^ (Figure 6c) display indefinite structures or sheet structures several hundred nanometers long. Although it is possible that both the irregular and layered structures were formed during sample preparation, as a result of evaporation on the carbon-coated Cu mesh, it is more likely that these nanostructures were randomly generated as a consequence of partial dissociation of polynuclear [Au(CN)_2_^−^]*_n_* complexes into shorter structures in association with random-coiled copolypeptides. Surprisingly, **2**/[Au(CN)_2_]^−^ (Figure 6b) exhibited numerous rectangular nanorods (length: 90–150 nm, width: 15–30 nm) together with some nanocrystals. These nanostructures are more developed than those of **1** and **3** and it is clear that the regular supramolecular structure resulting from **2** is due to the self-assembly of [Au(CN)_2_]^−^ in conjunction with the copolypeptide amphiphile. These results are consistent with the polynuclear behavior deduced from UV-vis, CD and luminescence spectral data.

**Figure 7:**
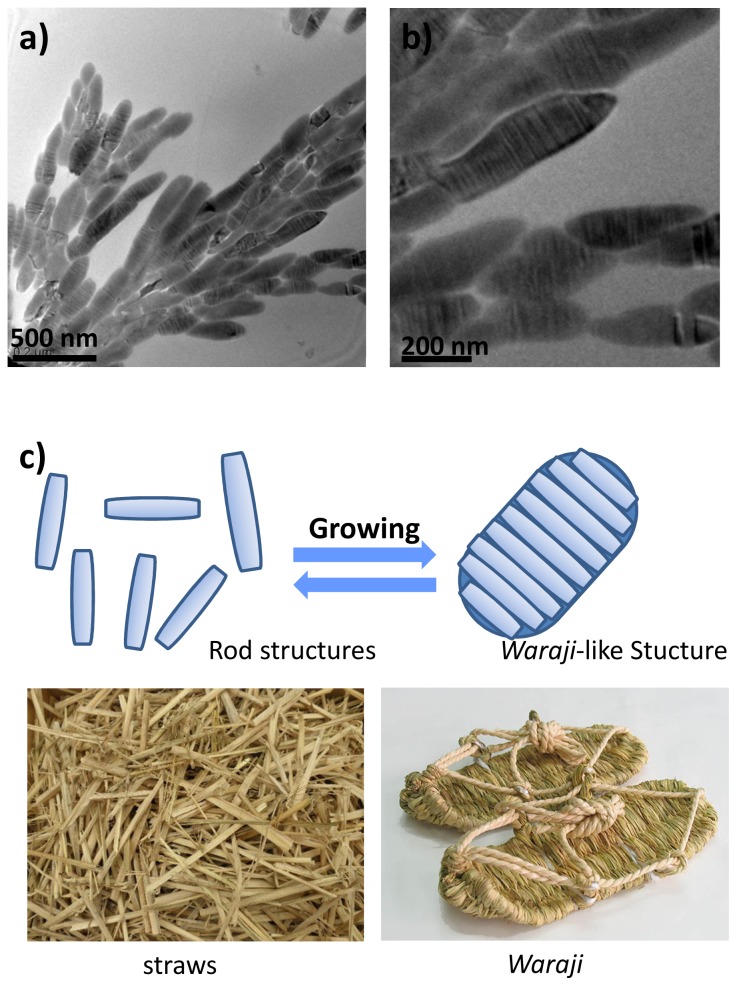
(**a**) Transmission electron micrograph of samples prepared from **2**/[Au(CN)_2_]^−^ ([Au(CN)_2_] = 5 mM (Lys units:K[Au(CN)_2_] = 1:1)). (**b**) Transmission electron micrographs of samples prepared from **2**/[Au(CN)_2_]^−^ ([Au(CN)_2_] = 5 mM (Lys units:K[Au(CN)_2_] = 1:1)) at higher magnification than micrograph (**a**). (**c**) Schematic illustration of rod structure to *Waraji* nanostructures comparing the Japanese *Waraji* (straw sandal) photographs.

When one molar equivalent of a 5 mM DI water solution of K[Au(CN)_2_] was added to a 5 mM (in terms of Lys units) DI water solution of **2**, a colloidal dispersion was obtained. Samples prepared from this solution displayed a nanostructure vaguely similar to the weave pattern of the traditional Japanese *Waraji* (straw sandals), with regular sections consisting of assembled nanorods 15–30 nm in length (Figure 7). This *Waraji*-like nanostructure presumably formed as the result of self-assembly of nanorods composed of the metal complex/amphiphile composite. It is known that diblock copolymer with a hydrophilic part and a hydrophibic part form one-directionally stacked lamellar structures due to the nucleation of lamellar layers, and the lamellar layers were anisotropically grown in the nanostructures [53,54]. Diblock copolypeptide amphiphiles and metal complexes therefore are capable of interacting not only at the molecular level but also at the subnanometer scale to form hierarchical structures, a phenomenon similar to the manner in which the quaternary structures of proteins are formed.

**Figure 8:**
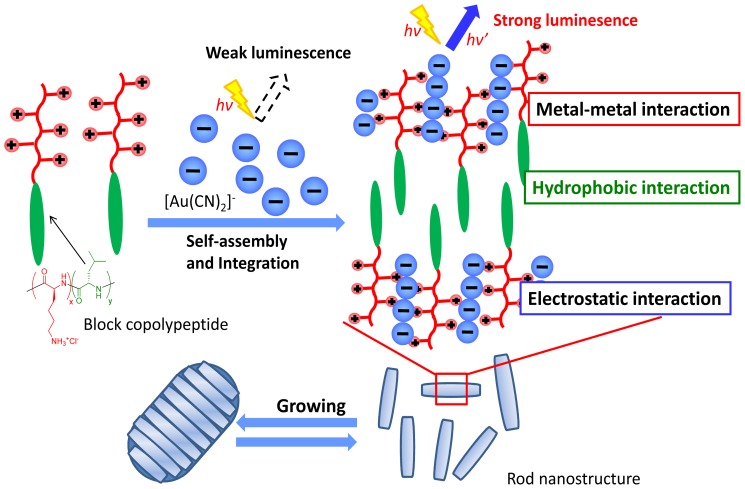
Schematic illustration of self-assembly of copolypeptide amphiphiles/Au complexes to develop the functional nanostructure.

The results from our spectroscopic and morphological investigations provide detailed information regarding the nature of the composites self-assembled from diblock copolypeptide amphiphiles and the metal complex, as illustrated in Figure 8. The data from UV-vis absorption and luminescence intensity analyses show that the amphiphile/[Au(CN)_2_]^−^ complexes include polynuclear [Au(CN)_2_]_n_ species which undergo Au-Au bonding interactions. In addition, based on the results obtained when using a molar ratio between the Lys component of the polypeptide and [Au(CN)_2_]^−^ of 1:1, the electrostatic interactions between the Lys segments and the anionic [Au(CN)_2_]^−^ play a significant role in enabling the aurophilic interactions. In particular, the observations concerning aurophilic interactions indicate that [Au(CN)_2_]^−^ forms ordered arrays together with the copolypeptide amphiphiles. High-resolution transmission electron microscopy (HR-TEM) observations show molecular-scale rod structures forming complex *Waraji*-like structures, depending on the concentration of the composite solution. It is therefore evident that the amphiphile is capable of inducing interesting alignment structures in aqueous solutions of the metal complex.

## 3. Experimental Section

### 3.1. Materials and Instrumentation

Tetrahydrofuran (THF) and hexane were dried by purging with nitrogen. Co(PMe_3_)_4_ was prepared according to procedures previously published in the literature. All chemicals were purchased from commercial suppliers (Tokyo Kasei, Wako Co., Ltd., Kanto Chemical Co., Ltd., and Sigma-Aldrich Chemical Co.) and used without further purification unless otherwise noted. Fourier Transform Infrared Spectroscopy (FTIR) measurements were obtained on a Spectrum 65 FT-IR (PerkinElmer, Inc.). ^1^H nuclear magnetic resonancy (NMR) spectra were acquired using an ESC 400 (JEOL Ltd.). Gel permeation chromatography/light scattering (GPC/LS) was performed at 333 K using a Waters high performance liquid chromatography (HPLC) system incorporating a 410 differential refractometer detector and a 1515 pump/controller. Separations were achieved using 10^5^, 10^4^ and 10^3^ Å Phenomenex Phenogel 5 μm columns with 0.1 M LiBr in DMF as the eluent and sample concentrations of 5 mg/mL. Pyrogen-free deionized water (DI) was obtained from Advantec RFD240NA and RFU655DA purification units. UV-vis spectra and fluorescence spectra were obtained on RF-2500PC and RF-5300PC spectrophotometers, respectively (Shimazu Co., Ltd.). Transmission electron microscopy was performed using a Tecnai G^2^ F20 (FEI Co.) operating at 200 kV. Transmission electron microscope samples were prepared by transferring the surface layers of gels or solutions to carbon-coated TEM grids.

### 3.2. General Polypeptide Synthesis

All diblock copolypeptide amphiphiles were synthesized using Co(PMe_3_)_4_ as the initiator, according to the literature procedures [44,45]. Protected diblock copolypeptide amphiphiles were first purified and then characterized using GPC/LS and FTIR. The protecting groups on the *N*_ɛ_-benzyloxycarbonyl-L-lisyne moieties were removed to produce residual L-lysine·HBr in the copolypeptides by the addition of 33 wt% HBr in acetic acid to a solution of copolymer in trifluoroacetic acid (TFA) at 273 K, followed by stirring for 1 h. All deprotected copolymers were dissolved in and then dialyzed exhaustively against non-pyrogenic DI water. Lyophilization of these solutions gave the copolymers as white powders. Chain lengths of Km segments were determined using GPC, with measured polydispersities (M_W_/M_n_) ranging from 1.13 to 1.19. ^1^H NMR in deuterium oxide (D_2_O) indicated over 99.9% removal of the benzyloxycarbonyl groups from the lysine residues.

### 3.3. General Preparation of Copolypeptide/[Au(CN)_2_]^−^ Composites

Ternary composites were prepared by mixing DI water solutions of K[Au(CN)_2_] (0.2 mM, 1 mL) with DI water solutions of diblock copolypeptide amphiphiles **1** to **3** (0.2 mM, 1 mL) at room temperature. All composite solutions prepared in this manner were stable in oxygen-free DI water for a period of one month, although pure K[Au(CN)_2_] in DI water was unstable in air and decomposed with the formation of both oxidation and hydrolysis products, with a concurrent loss of photoluminescence. The observed stabilization induced on adding the amphiphiles indicates that aggregation of the composites subsequent to the copolypeptides addition prevents [Au(CN)_2_]^−^ from reacting with oxygen (see Section 2.3).

## 4. Conclusions

We have demonstrated the formation of diblock copolypeptide amphiphile/metal complex composites, with significant variation in nanostructure depending on the structure of the copolypeptide. The formation of composite materials produced by combining these amphiphiles with the metal complex demonstrates that it is possible to control the complex’s metal-metal interactions and produce one-dimensional structures such as rods, as well as more complex architectures such as the *Waraji* structure. The technique of combination of amiphiphilic molecules and discrete coodication compounds [55,56] makes it possible to design flexible, reversible, and signal-responsive supramolecular coordination systems. This general concept of a biopolymer composite could be expanded to include other useful compounds and should provide valuable information leading to further advances in the field of coordination materials and biopolymer nanochemistry.
